# Mosquitoes Put the Brake on Arbovirus Evolution: Experimental Evolution Reveals Slower Mutation Accumulation in Mosquito Than Vertebrate Cells

**DOI:** 10.1371/journal.ppat.1000467

**Published:** 2009-06-05

**Authors:** Nikos Vasilakis, Eleanor R. Deardorff, Joan L. Kenney, Shannan L. Rossi, Kathryn A. Hanley, Scott C. Weaver

**Affiliations:** 1 Center for Biodefense and Emerging Infectious Diseases and Department of Pathology, University of Texas Medical Branch, Galveston, Texas, United States of America; 2 Department of Biology, New Mexico State University, Las Cruces, New Mexico, United States of America; University of California San Francisco, United States of America

## Abstract

Like other arthropod-borne viruses (arboviruses), mosquito-borne dengue virus (DENV) is maintained in an alternating cycle of replication in arthropod and vertebrate hosts. The trade-off hypothesis suggests that this alternation constrains DENV evolution because a fitness increase in one host usually diminishes fitness in the other. Moreover, the hypothesis predicts that releasing DENV from host alternation should facilitate adaptation. To test this prediction, DENV was serially passaged in either a single human cell line (Huh-7), a single mosquito cell line (C6/36), or in alternating passages between Huh-7 and C6/36 cells. After 10 passages, consensus mutations were identified and fitness was assayed by evaluating replication kinetics in both cell types as well as in a novel cell type (Vero) that was not utilized in any of the passage series. Viruses allowed to specialize in single host cell types exhibited fitness gains in the cell type in which they were passaged, but fitness losses in the bypassed cell type, and most alternating passages, exhibited fitness gains in both cell types. Interestingly, fitness gains were observed in the alternately passaged, cloned viruses, an observation that may be attributed to the acquisition of both host cell–specific and amphi-cell-specific adaptations or to recovery from the fitness losses due to the genetic bottleneck of biological cloning. Amino acid changes common to both passage series suggested convergent evolution to replication in cell culture via positive selection. However, intriguingly, mutations accumulated more rapidly in viruses passed in Huh-7 cells than in those passed in C6/36 cells or in alternation. These results support the hypothesis that releasing DENV from host alternation facilitates adaptation, but there is limited support for the hypothesis that such alternation necessitates a fitness trade-off. Moreover, these findings suggest that patterns of genetic evolution may differ between viruses replicating in mammalian and mosquito cells.

## Author Summary

Dengue virus (DENV) is an arthropod-borne virus of immense public health concern. Recent studies suggest that the rate of DENV evolution has accelerated, as well as the disease severity (virulence), most likely driven by the increased transmission and selection by the vector, *Aedes aegypti*, over the last 50 years. Despite the nearly global impact of DENV, little is known about the forces that influence its host range evolution, a deficiency confounded by the absence of a practical vertebrate model. We used vertebrate (Huh-7) and invertebrate (C6/36) cells lines as surrogate hosts for studying DENV host range evolution. Using genetically homogeneous or heterogeneous DENV for serial passages (specialization) in either cell line, or alternating passage, we demonstrated that (i) viruses allowed to specialize in single host cells exhibited fitness gains but fitness losses in the bypassed cell line; (ii) most viruses passaged in alternating cycles exhibited detectable fitness gains in both cell lines; (iii) amino acid changes common in both passage series suggested convergent evolution via positive selection; and (iv) there was limited support that DENV evolution is constrained by alternate host transmission and replication. These results provide insights into the likelihood and extent of shifts towards higher DENV virulence.

## Introduction

Arthropod-borne RNA viruses (arboviruses) pose an especially high risk of emergence from reservoir hosts into humans [1],[2], due to the genetic plasticity of the viral RNA genome coupled with the dispersal potential of arthropod vectors and some amplification hosts. Paradoxically, vector-borne RNA viruses undergo substantially slower rates of evolution (by a factor of ten) than many of their directly-transmitted counterparts [3],[4],[5]. To explain this disparity, the trade-off hypothesis postulates that alternating replication in vertebrate and arthropod hosts constrains arbovirus evolution, because a fitness increase in one host usually diminishes fitness in the other.

Experimental evolution *in vivo* has been a valuable approach for testing this hypothesis. In particular, it has been illuminating to compare the evolution of viruses that have been serially passaged in a single host versus viruses passaged in alternation between two different hosts. For example, Ross River Virus, an alphavirus, showed phenotypic stability during alternating passages in *Aedes aegypti* mosquitoes and mice but increased in neurovirulence after serial passage in mouse brains, suggesting that host alternation restrains the evolution of virulence [6],[7]. Similarly, in another alphavirus, Venezuelan equine encephalitis (VEEV), lineages passaged 10 times in *Ae. aegypti* exhibited an increase in mosquito infectivity relative to viruses passaged in alternation between rodents and mosquitoes, while rodent-specialized strains produced higher viremias in rodents relative to alternately-passaged virus. Moreover both serially-passaged VEEV lines exhibited fitness declines in the bypassed host, while lineages passaged in mosquitoes and rodents in alternation demonstrated no detectable fitness gains, or losses, in either mosquitoes or vertebrates [8].

Experimental evolution *in vitro*, while subject to particular caveats discussed below, has also yielded valuable insights into the constraints that shape arbovirus evolution. Studies of serially-passaged Sindbis virus (SINV) confirm fitness gains in single host-adapted (specialized) viruses in the cell substrate used for passage, and fitness losses in the bypassed cell line, as well as fitness gains in most alternately passaged viruses [9]. Similar results were observed with eastern equine encephalitis virus (EEEV) passaged in mammalian and mosquito cell lines [10] or avian and mosquito cell lines [11]. Surprisingly, EEEV passaged in alternation increased in fitness in both cell types, in a manner similar to that observed for single host-adapted viruses, but showed a lower rate of mutation than single-host adapted viruses [11]. However, studies with vesicular stomatitis virus (VSV) do not support the hypothesis that alternating host replication imposes fitness trade-offs, as symmetrical fitness increases were observed in both single host-adapted (specialized) and alternate-passaged viruses [12]. Nonetheless, in another study VSV was allowed to specialize in a vertebrate cell host [baby hamster kidney cells (BHK)] and it exhibited substantial fitness gains for those cells but fitness declines on novel host (HeLa or MDCK) cells [13].

Mosquito-borne dengue virus (DENV) offers an especially interesting system in which to examine the trade-off hypothesis for 3 major reasons. First, the 4 serotypes of endemic DENV that circulate between humans and peridomestic *Aedes* mosquitoes are known to have emerged in four independent events from sylvatic progenitors that are maintained in non-human primates and arboreal *Aedes* spp. [14]. Today, these endemic DENV lineages are both ecologically and evolutionarily independent from the ancestral sylvatic cycles. It is therefore possible to compare the patterns of, and constraints on, the evolution of viral isolates from both the ancestral and derived transmission cycles. Second, phylogenetic studies indicate that the rate of evolution in endemic DENV has accelerated substantially [3],[15],[16],[17], probably driven by the surge in transmission that has occurred over the last 50 years [18]. Since there is neither a vaccine nor licensed antiviral therapy available to control DENV spread, it is increasingly important to gain insights into the factors that will shape its evolution as the pandemic progresses. Finally, it is possible that DENV is under selection for increasing virulence. Severe disease [dengue hemorrhagic fever/dengue shock syndrome (DHF/DSS)] was first documented in the 1950's and has dramatically increased in frequency ever since [19],[20],[21],[22]. While sequential infection by two different DENV serotypes is a major risk factor for severe disease [23],[24],[25],[26],[27], it has also been shown that individual DENV strains and genotypes vary in their tendency to cause severe disease [20],[21],[22],[28],[29],[30],[31]. Moreover, there are now several examples of DENV strains associated with severe disease displacing milder strains [32],[33]. For example, extensive epidemiological and phylogenetic evidence indicates that Southeast Asian DENV-2 genotypes are more likely to cause DHF/DSS than American or South Pacific genotypes. After it was introduced into the Americas, the Southeast Asian DENV-2 genotype began to displace the native American genotype [34],[35],[36],[37],[38],[39],[40],[41],[42],[43]. Studies of DENV evolution could provide insight into the likelihood and extent of shifts to higher virulence.

**Figure 1:**
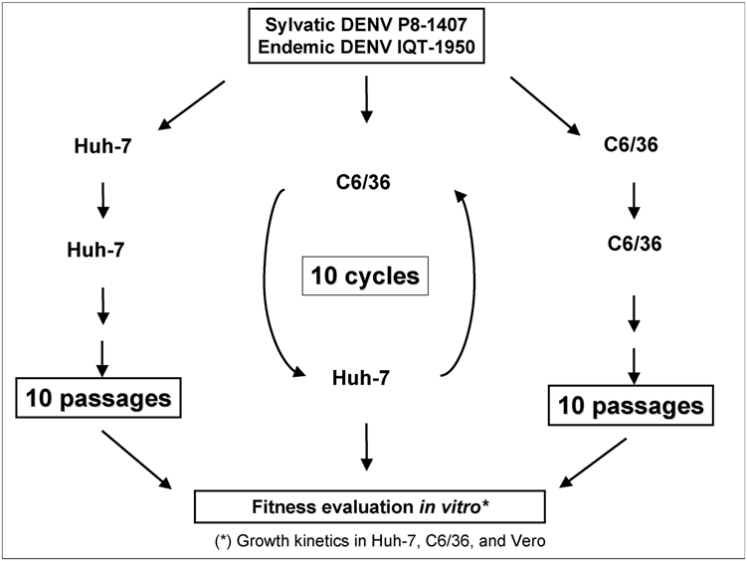
Experimental design for DENV *in vitro* adaptation studies. Sylvatic strain P8-1407 or endemic strain IQT-1950 were serially passaged in vertebrate Huh-7 cells (left) or invertebrate C6/36 cells (*Ae. albopictus* cell line) (right) to artificially bypass one host, or alternately passaged (center) to simulate natural transmission. The fitness of DENV derived from these passage series compared to parent viruses was determined by direct replication comparison.

Three hypotheses were tested in the current study: (i) releasing DENV from alternating host replication by repeated passages in a single host facilitates adaptation to that host and results in an increase in mutation accumulation; (ii) adaptation to a single host is usually specific and results in fitness declines in other hosts; and (iii) host alternation selects for virus populations that are genetically conserved and show little or no adaptation to either host. To test these hypotheses, one isolate of endemic and one isolate of sylvatic DENV-2 were maintained in one of 3 serial passage regimens: 10 passages in human vertebrate cells; 10 passages in mosquito cells, or 10 alternating passages between the two cells lines (10 passages in each cycle; 20 passages total) (Figure 1). In the absence of an established and practical *in vivo* vertebrate model for studying DENV host range evolution, we used the human hepatoma Huh-7 and *Ae. albopictus* C6/36 cell lines as surrogate hosts for host range evolution. The rationale for utilizing the Huh-7 cell line lies in the *in vivo* liver involvement in DENV infection [44],[45],[46], and *Ae. albopictus* is an important secondary DENV vector [18]. The fitness of each passaged series was compared to that of other evolved lineages and to the parent virus in both the cell lines used for passage, the bypassed cell line and a novel cell line (African green monkey kidney Vero cells) that was not utilized in any of the passage series. To assess the impact of quasispecies diversity on patterns of adaptation, passages of each strain were initiated separately, with either a cloned (plaque-purified biological clone) virus that was genetically relatively homogeneous or uncloned viruses that encompassed a more diverse mutant spectrum. The definition and utility of the term ‘quasispecies’ has recently been questioned [47],[48],[49],[50]. For the purpose of our work, we define quasispecies as the intra-host viral genetic variation that arises due to the high error rate of the viral polymerase. Cloned viruses additionally allowed us to determine whether cell-specific fitness gains in evolved viruses were likely the result of specific mutations.

## Results

One important consideration for studies of virus evolution *in vitro* is that viruses may adapt to features of cultured cells that are irrelevant *in vivo*, such as temperature [12],[51], defective interfering (DI) particles, or overexpression of heparan sulfate (HS) [52],[53],[54]. To overcome the former two limitations, all infected cell cultures were incubated at 32°C and a low multiplicity of infection was maintained to minimize the evolution of defective interfering particles. While it was not possible to influence the expression of HS in the cells used, we utilized duplicate parallel passage series for each treatment to assess variance in outcomes Additionally, viral replication curves show inherent variability due to: (i) the dependence of virus replication on inoculum titers, which results in inaccuracies of replication estimates and (ii) underestimation of the cell-adapted (i.e. Huh-7) virus titer due to loss of the ability to form visible plaques in the cell line where progeny virus titer is evaluated (e.g. C6/36). Previous studies have used competition assays with genetic markers to overcome this latter limitation [8],[9],[10]. However the lack of an infectious clone for sylvatic DENV-2 prevented the use of genetic markers. Instead, focus-forming assays were used to detect expressed antigen on the surface of infected cells minimizing the possibility for underestimation of the cell-adapted virus titers.

### Comparisons of fitness and general patterns of adaptation

**Figure 2:**
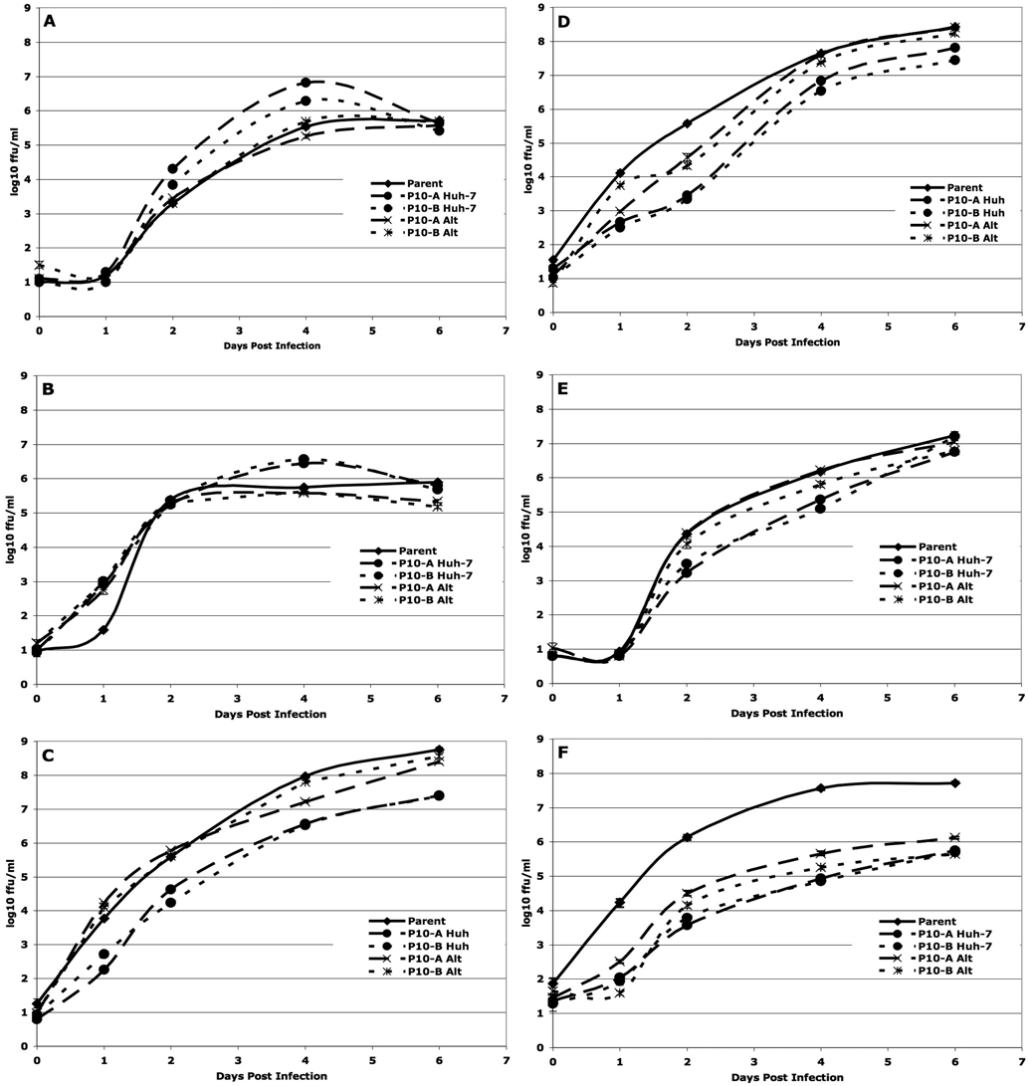
Replication of Huh-7 – passaged uncloned DENV-2. (a) Huh-7 – passaged sylvatic P8-1407 DENV-2 on Huh-7 cells. (b) Huh-7 – passaged endemic IQT-1950 DENV-2 on Huh-7 cells. (c) Huh-7 – passaged sylvatic P8-1407 DENV-2 on the bypassed cell line C6/36. (d) Huh-7 – passaged endemic IQT-1950 DENV-2 on the bypassed cell line C6/36. (e) Huh-7 – passaged sylvatic P8-1407 DENV-2 on a control cell line (Vero). (f) Huh-7 – passaged endemic IQT-1950 DENV-2 on a control cell line (Vero). Timepoint T = 0 represents residual virus after washing.

**Figure 3:**
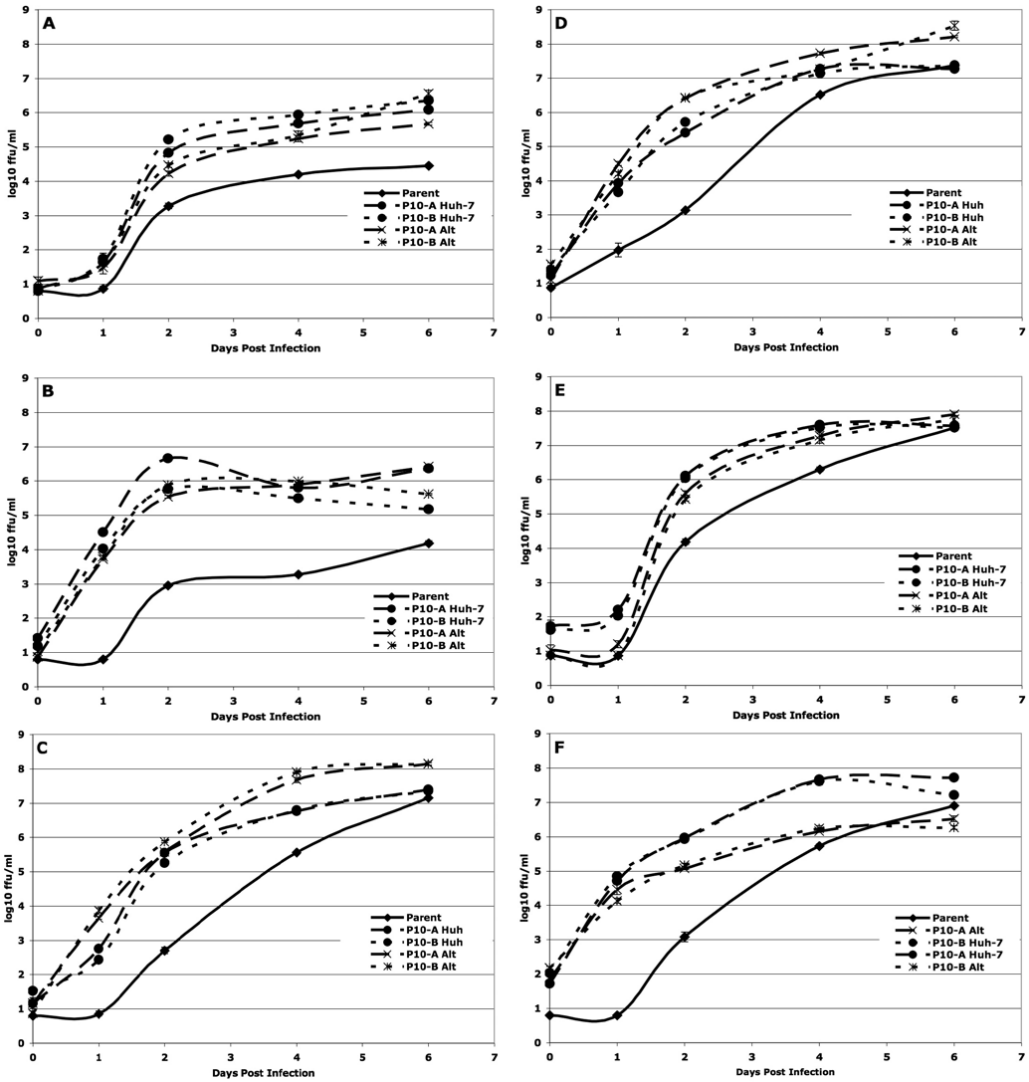
Replication of Huh-7 – passaged cloned DENV-2. (a) Huh-7 – passaged sylvatic P8-1407 DENV-2 on Huh-7 cells. (b) Huh-7 – passaged endemic IQT-1950 DENV-2 on Huh-7 cells. (c) Huh-7 – passaged sylvatic P8-1407 DENV-2 on the bypassed cell line C6/36. (d) Huh-7 – passaged endemic IQT-1950 DENV-2 on the bypassed cell line C6/36. (e) Huh-7 – passaged sylvatic P8-1407 DENV-2 on a control cell line (Vero). (f) Huh-7 – passaged endemic IQT-1950 DENV-2 on a control cell line (Vero). Timepoint T = 0 represents residual virus after washing.

**Figure 4:**
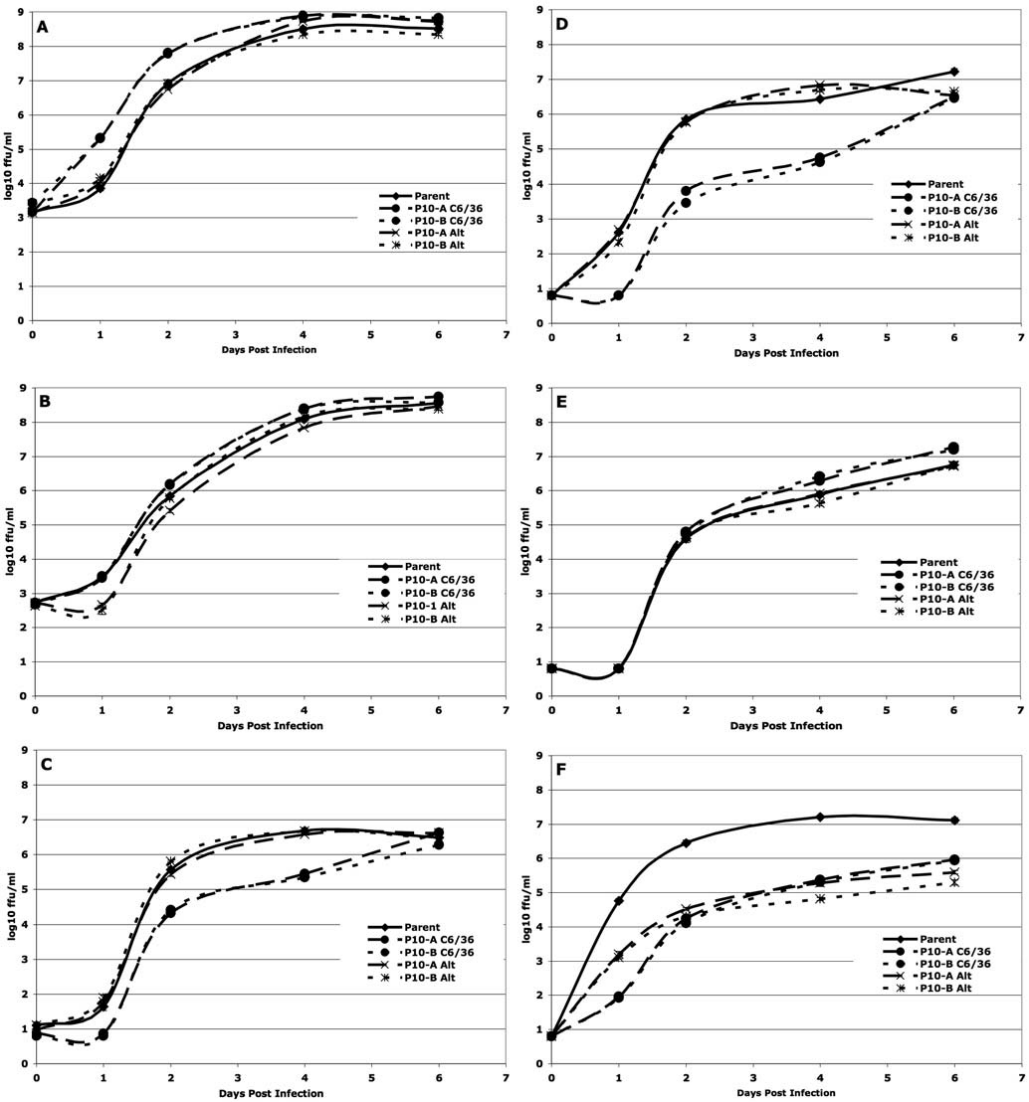
Replication of C6/36 – passaged uncloned DENV-2. (a) C6/36 – passaged sylvatic P8-1407 DENV-2 on C6/36 cells. (b) C6/36 – passaged endemic IQT-1950 DENV-2 on C6/36 cells. (c) C6/36 – passaged sylvatic P8-1407 DENV-2 on the bypassed cell line Huh-7. (d) C6/36 – passaged endemic IQT-1950 DENV-2 on the bypassed cell line Huh-7. (e) C6/36 – passaged sylvatic P8-1407 DENV-2 on a control cell line (Vero). (f) C6/36 – passaged endemic IQT-1950 DENV-2 on a control cell line (Vero). Timepoint T = 0 represents residual virus after washing.

**Figure 5:**
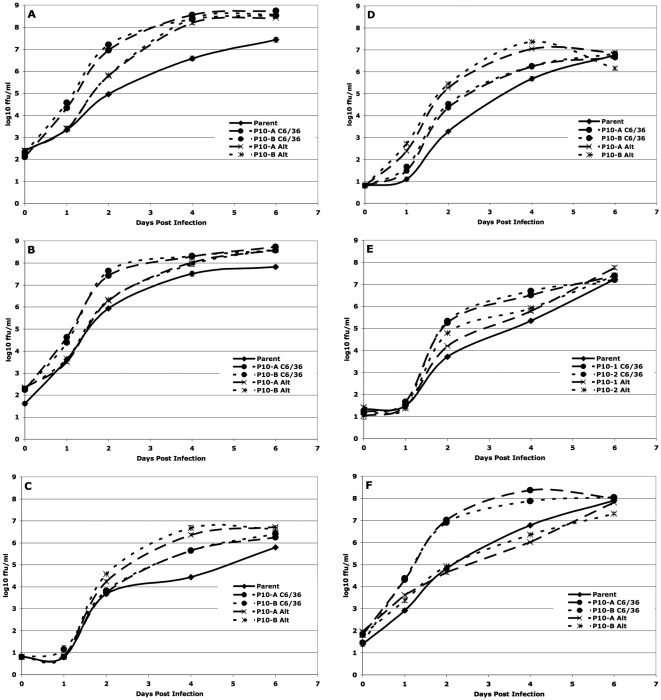
Replication of C6/36 – passaged cloned DENV-2. (a) C6/36 – passaged sylvatic P8-1407 DENV-2 on C6/36 cells. (b) C6/36 – passaged endemic IQT-1950 DENV-2 on C6/36 cells. (c) C6/36 – passaged sylvatic P8-1407 DENV-2 on the bypassed cell line Huh-7. (d) C6/36 – passaged endemic IQT-1950 DENV-2 on the bypassed cell line Huh-7. (e) C6/36 – passaged sylvatic P8-1407 DENV-2 on a control cell line (Vero). (f) C6/36 – passaged endemic IQT-1950 DENV-2 on a control cell line (Vero). Timepoint T = 0 represents residual virus after washing.

As described in the Methods section, the replication dynamics of a given DENV population serially passaged in a single cell line was compared only to the matched population passaged in alternation and to the parent population, using a repeated measures analysis of variance (rmANOVA). The rmANOVA compares the shape and height of the replication curve, thereby incorporating both the rate and maximum level of viral replication, hereafter referred to as “fitness”. The individual panels in Figures 2, 3, 4, and 5 show the patterns of replication in each of these comparison groups graphically, while Tables 1 and 2 summarize the statistical comparisons of fitness. For example, the statistical comparison of the replication curves shown in Figure 2A are represented in the first column of Table 1.

**Table 1:** Comparison of the replication kinetics of individual lineages within an experimental group including the parent virus (Parent), duplicate lineages passaged exclusively in Huh-7 cells (P10 Huh-7) or duplicate lineages passaged in alternation between Huh-7 and C6/36 cells (P10 Alt).

	Significance of Comparisons Between Lineages Within Experimental Groups*											
Virus strain:	P8-1407		IQT-1950		P8-1407		IQT-1950		P8-1407		IQT-1950	
Population type:	Uncloned	Cloned	Uncloned	Cloned	Uncloned	Cloned	Uncloned	Cloned	Uncloned	Cloned	Uncloned	Cloned
Cell substrate for replication curve:	HuH-7	HuH-7	HuH-7	HuH-7	Vero	Vero	Vero	Vero	C6/36	C6/36	C6/36	C6/36
P10-A H7 v P10-B H7	↑						↓				↑	
P10-A Alt v P10-B Alt	↓	↓			↑	↑	↑					
P10-A H7 v Parent	↑	↑	↑	↑	↓	↑	↓	↑	↓	↑	↓	↑
P10-B H7 v Parent	↑	↑	↑	↑	↓	↑	↓	↑	↓	↑	↓	↑
P10-A Alt v Parent		↑		↑		↑	↓	↑	↓	↑	↓	↑
P10-B Alt v Parent	↑	↑		↑	↓	↑	↓	↑		↑	↓	↑
P10-A H7 v P10-A Alt	↑	↑	↑	↑	↓	↑	↓	↑	↓	↓	↓	↓
P10-A H7 v P10-B Alt	↑		↑	↑	↓	↑		↑	↓	↓	↓	↓
P10-B H7 v P10-A Alt	↑	↑	↑	↓	↓	↑	↓	↑	↓	↓	↓	↓
P10-B H7 v P10-B Alt		↑	↑	↓	↓	↑	↑	↑	↓	↓	↓	↓

*: An arrow in a cell indicates a significant difference between two lineages based on a Tukey-Kramer post-hoc comparison, an empty cell indicates that no significant difference was detected between the lineages. The direction of the arrow indicates the overall progeny production of the first virus in the comparison versus the second virus. Experimental Groups are defined by the virus used to initiate passages, the population of virus, consensus or cloned, used to initiate passages, and the cell line in which the replication curve was conducted.

**Table 2:** Comparison of the replication kinetics of individual lineages within an experimental group, including the parent virus (Parent), duplicate lineages passaged exclusively in C6/36 cells (P10 C6/36) or duplicate lineages passaged in alternation between Huh-7 and C6/36 cells (P10 Alt).

	Significance of Comparisons Between Lineages Within Experimental Groups*											
Virus strain:	P8-1407		IQT-1950		P8-1407		IQT-1950		P8-1407		IQT-1950	
Population type:	Uncloned	Cloned	Uncloned	Cloned	Uncloned	Cloned	Uncloned	Cloned	Uncloned	Cloned	Uncloned	Cloned
Cell substrate for replication curve:	C6/36	C6/36	C6/36	C6/36	Vero	Vero	Vero	Vero	H7	H7	H7	H7
P10-A C6/36 v P10-B C6/36								↑			↑	↓
P10-A Alt v P10-B Alt							↑					
P10-A C6/36 v Parent	↑	↑	↑	↑	↑	↑	↓	↑	↓	↑	↓	↑
P10-B C6/36 v Parent	↑	↑	↑	↑	↑	↑	↓	↑	↓	↑	↓	↑
P10-A Alt v Parent		↑	↓	↑		↑	↓			↑		↑
P10-B Alt v Parent		↑	↓	↑		↑	↓			↑	↓	↑
P10-A C6/36 v P10-A Alt	↑	↑	↑	↑	↑	↑	↑	↑	↓	↓	↓	↑
P10-A C6/36 v P10-B Alt	↑	↑	↑	↑	↑	↑		↑	↓	↓	↓	↑
P10-B C6/36 v P10-A Alt	↑	↑	↑	↑	↑	↑	↓	↑	↓	↓	↓	↑
P10-B C6/36 v P10-B Alt	↑	↑	↑	↑	↑	↑		↑	↓	↓	↓	↑

*: An arrow in a cell indicates a significant difference between two lineages based on a Tukey-Kramer post-hoc comparison, an empty cell indicates that no significant difference was detected between the lineages. The direction of the arrow indicates the overall progeny production of the first virus in the comparison versus the second virus. Experimental Groups are defined by the virus used to initiate passages, the population of virus, uncloned or cloned, used to initiate passages, and the cell line in which the replication curve was conducted.

Overall, the data from this study, which are described in detail below, reveal several general trends in the adaptation of sylvatic and endemic DENV during exclusive and alternating passage. First, cloned viruses showed a decline in fitness relative to the uncloned parent tested immediately following plaque purification. Subsequently, cloned viruses passaged on any cell type showed a dramatic gain in fitness in all cell types. In contrast, passage of uncloned viruses in single cell type (Huh-7 or C6/36) resulted in increased fitness in that cell type, and loss of fitness in the bypassed cell type. Passage of the endemic virus in alternation did not affect fitness in Huh-7 cells, but decreased fitness in C6/36 cells and in Vero cells, a novel host cell. However passage of the sylvatic virus in alternation had little effect on fitness in any cell type. Finally, passage in Huh-7 cells reliably led to a decrease in fitness in Vero cells, but passage in C6/36 resulted in variable changes in fitness (increases and decreases) in Vero cells.

### Fitness of viruses passaged in a vertebrate cell line serial passage and alternating passage of uncloned DENV

In general, fitness changes of duplicate DENV-2 lineages were similar to one another (Tables 1 and 2). Reading horizontally across the top two rows, note the general lack of arrows (lack of significance) for the comparison of the A and B replicates of a given passage series. Exclusive passage in Huh-7 resulted in an increase in fitness in this cell line, relative to the parent, of both the sylvatic (P8-1407) and endemic (IQT-1950) strains. In contrast, alternating passage, with the exception of uncloned sylvatic strain P8-1407 P10-B), usually had no impact on fitness in Huh-7 cells (Figures 2A, B; Table 1).

To determine whether increases in fitness in the cell line used for passage (Huh-7 cells) came at the cost of decreased replication in the bypassed cell lines, replication curves were also evaluated in C6/36 cells. Fitness of both Huh-7-adapted endemic and sylvatic DENV strains was significantly lower than those of the parents and alternating passage viruses in C6/36 cells (Figures 2C, D), indicating that adaptation was host cell-specific.

To determine whether increases in fitness in the cell line used for passage came at the cost of decreased replication in additional cell lines, replication curves were also evaluated in a novel cell line, Vero cells. Fitness of both Huh-7-adapted endemic and sylvatic DENV strains was significantly lower than those of the parents and alternately passage viruses in Vero cells (Figures 2E, F). Interestingly, the fitness of the alternately passaged viruses was also significantly lower than the parent virus in most lineages (Figure 2E, F). These results, summarized in Table 1, also indicate that adaptation was cell-specific.

### Serial passage and alternating passage of cloned DENV

In general, relative to the parental viruses, both biologically cloned DENV strains showed increases in fitness after either specialized or alternating passage series. This pattern is likely driven by the initial loss in fitness that followed generation of the clonal populations: the cloned sylvatic parent's fitness was significantly less than the uncloned parent (Figure 2A) both 4 (5.53±0.07 vs 4.2±0.03 log_10_ ffu/ml) and 6 days p.i. (5.73±0.03 vs 4.45±0.05 log_10_ ffu/ml) and similarly, the cloned sylvatic parent's fitness was less than that of its uncloned parent both 4 (5.75±0.02 vs 3.28±0.03 log_10_ ffu/ml) and 6 days p.i. (5.9±0.02 vs 4.19±0.06 log_10_ ffu/ml) (Figure 2B). This significant reduction of the cloned parent's replication may be attributable to sequence differences compared to the uncloned viruses, since the consensus sequence (most common nucleotide at each position for the virus population) of the initial cloned populations differed from the uncloned populations (Table 3). Similar reductions in fitness of biological clones compared to the parental population have been reported for VSV [55].

**Table 3:** Sequence differences between uncloned (parent) and cloned IQT-1950 and P8-1407 viruses.

DENV strain	Nucleotide changes during plaque cloning	Amino acid change	Genome region/Gene
P8-1407	C2535T	-	NS1
P8-1407	C4010T	S→L	NS2A
P8-1407	C4902T	-	NS3
P8-1407	T6579A	-	NS4A
P8-1407	T9909C	T→N	NS5
P8-1407	C9986A	-	NS5
IQT-1950	C6359T	-	NS3
IQT-1950	T10392C	NA	3′ UTR

- = no change; NA = not applicable.

Fitness of the cloned sylvatic and endemic strains passaged exclusively in Huh-7 cells was significantly higher (P<0.001) than those of the parent when their replication was evaluated in Huh-7 cells (Figures 3A, B). The fitness of both endemic and sylvatic alternately passaged viruses was significantly higher than those of their parents, and the overall fitness of the specialized and alternately passaged viruses did not differ significantly. Similarly, fitness in the bypassed C6/36 cell line of both alternately passaged DENV-2 strains was significantly higher than for the parent virus, although in the Huh-7 – specialized viruses fitness was significantly lower than for the alternately passaged viruses (Figures 3C, D). In Veros, a novel cell line, fitness of both endemic and sylvatic Huh-7-adapted DENV strains was higher than for the parents and alternately passaged lineages (Figure 3E, F). As summarized in Table 1, biologically cloned viruses exhibited a different pattern of adaptation than uncloned viruses, with adaptation showing a less specialized pattern, a reflection of the inherent low fitness of the cloned viruses.

### Fitness of viruses passaged in mosquito cells serial and alternating passage of uncloned DENV

In general, fitness of duplicate mosquito cell-specialized lineages was similar (Table 2). Exclusive passage in C6/36 cells resulted in an increase in fitness, relative to the parent, for the uncloned isolates of both the sylvatic (P8-1407) and endemic (IQT-1950) DENV strains. In contrast, alternating passage did not result in an increase in fitness, relative to the parent, for the uncloned isolates of the sylvatic DENV strain (Figure 4A), and resulted in a decrease in fitness for the endemic DENV strain (Figure 4B).

To determine whether increases in fitness in the cell line used for passage came at the cost of decreased replication in other cell lines, replication kinetics were also evaluated in the bypassed Huh-7 and Vero cells. When their replication was evaluated in Huh-7 cells, fitness of both C6/36-adapted endemic and sylvatic DENV strains was significantly lower than for the parents and alternately passage viruses (Figure 4C, D). Both parent and alternately passaged viruses had similar replication kinetics. Surprisingly, when their replication was evaluated in Vero cells, fitness of the C6/36-specialized sylvatic strain was significantly higher than that of the parents; no fitness changes were observed for the alternating passage viruses (Figure 4E). Fitness of the C6/36-adapted and alternate passage endemic strain was significantly lower than for the parent (Figure 4F). Overall, the data suggest that exclusive passage of viruses in C6/36 cells improves fitness in C6/36 cells, either improves or decreases fitness in the control Vero cell line and decreases fitness in the bypassed Huh-7 cell line, whereas the alternating passage either has no effect or predominantly fitness costs everywhere.

### Serial and alternating passage of biologically cloned DENV

As with the biologically cloned populations passaged in Huh-7 cells, the fitness of cloned viruses passaged in C6/36 cells showed a general pattern of increase relative to the parent and the fitness of duplicate lineages was similar (Table 2). Both specialized C6/36 and alternating passages resulted in an increase in fitness in the C6/36 cell environment, relative to the parent, for the cloned isolates of both the sylvatic and endemic DENV strains (Figure 5A, B).

To determine whether fitness increases that occurred in the cell line used for passage came at the cost of decreased replication in other cell lines, replication kinetics were also evaluated in the bypassed cell lines (Huh-7 and Vero). In Huh-7 cells, fitness of both C6/36 – adapted endemic and sylvatic DENV strains were significantly lower than those of the alternately passaged viruses [but the replication kinetics of parent viruses were significantly lower than both adapted lineages (Figures 5C, D)]. Fitness of both C6/36 – adapted sylvatic and endemic strains was higher than for the parents and alternating passage series (except for the endemic where there was no fitness increase) when their replication was evaluated in Vero cells (Figure 5E, F). These results indicate that adaptation to mosquito cells was not entirely cell-specific and are summarized in Table 2.

### Consensus sequence changes of uncloned DENV

The consensus genomic sequences of the 10^th^ serial passage in Huh-7 and C6/36 cells, and the final alternating series (which terminated in Huh-7 cells) were compared to the parental DENV sequences. In both alternating passage series combined, sylvatic strain P8-1407 accrued 11 consensus mutations distributed throughout the genome. Five mutations presented as nucleotide mixtures (a mixture of two nucleotides, parental and mutant, were indicated on sequence electropherograms of both cDNA strands) and 3 of the 11 mutations were synonymous (Table 4). When the same strain was allowed to specialize in Huh-7 cells for 10 consecutive passages, it accrued 10 consensus mutations, also distributed throughout the genome, of which 4 were nucleotide mixtures and 6 were synonymous (Table 4). Endemic strain IQT-1950 accrued 12 consensus mutations after 10 alternating passages; five mutations presented as nucleotide mixtures and 6 of the 12 accrued mutations were synonymous (Table 5). When the same DENV strain was allowed to specialize in Huh-7 cells, it accrued 10 consensus mutations of which 5 presented as nucleotide mixture and 5 were synonymous (Table 5).

**Table 4:** Nucleotide (nt) and amino acid (aa) changes in sequences of alternating and Huh-7 – passaged strain P8-1407 DENV relative to uncloned parent P8-1407.

Virus	Passage^a^	nt position	Region	nt change	aa change
P8-1407	P10 – A Alt	325	Cap	G→R (G/A)	(A→T)
P8-1407	P10 – B	1120	ENV	G→R (G/A)	(E→K)
P8-1407	P10 – A Alt	1120	ENV	G→R (G/A)	(E→K)
P8-1407	P10 – B Alt	1120	ENV	G→A	E→K
P8-1407	P10 – B Alt	1405	ENV	A→G	K→E
P8-1407	P10 – A	1540	ENV	G→A	E→K
P8-1407	P10 – B	1673	ENV	A→W (A/T)	(K→M)
P8-1407	P10 – B Alt	2720	NS1	A→G	-
P8-1407	P10 – B	2722	NS1	A→R (A/G)	K→E
P8-1407	P10 – B Alt	2739	NS1	C→T	K→E
P8-1407	P10 – A	4378	NS2B	A→R (A/G)	(S→G)
P8-1407	P10 – A	4902	NS3	C→T	-
P8-1407	P10 – A	6579	NS4A	T→A	-
P8-1407	P10 – B Alt	7724	NS5	A→C	H→P
P8-1407	P10 – B	9179	NS5	G→C	G→A
P8-1407	P10 – A Alt	9179	NS5	G→S (G/C)	(G→A)
P8-1407	P10 – B Alt	9179	NS5	G→C	G→A
P8-1407	P10 – A Alt	9186	NS5	C→Y (C/T)	-
P8-1407	P10 – A Alt	9276	NS5	C→Y (C/T)	-
P8-1407	P10 – A Alt	9746	NS5	T→C	-
P8-1407	P10 – A	9987	NS5	T→C	-
P8-1407	P10 – B	10400	3′- UTR	T→C	-
P8-1407	P10 – A Alt	10400	3′- UTR	T→C	-
P8-1407	P10 – B Alt	10400	3′- UTR	T→C	-

a Each passage was performed in duplicate (series A and B); P10 = passage 10; Alt = alternate passage.

**Table 5:** Nucleotide (nt) and amino acid (aa) changes in sequences of Huh-7 – passaged IQT-1950 strain DENV relative to uncloned parent DENV.

Virus	Passage^a^	nt position	Region	nt change	aa change
IQT-1950	P10 – A	506	prM	T→Y (T/C)	-
IQT-1950	P10 – B Alt	978	ENV	G→S (G/C)	(V→L)
IQT-1950	P10 – A	1119	ENV	G→A	E→K
IQT-1950	P10 – B	1121	ENV	G→A	-
IQT-1950	P10 – A Alt	1121	ENV	G→R (G/A)	-
IQT-1950	P10 – A Alt	1412	ENV	T→Y (T/C)	-
IQT-1950	P10 – A	1539	ENV	G→R (G/A)	(E→K)
IQT-1950	P10 – B	1539	ENV	G→A	E→K
IQT-1950	P10 – A Alt	1539	ENV	G→A	E→K
IQT-1950	P10 – B Alt	1539	ENV	G→A	E→K
IQT-1950	P10 – B	1657	ENV	A→G	-
IQT-1950	P10 – B Alt	2014	ENV	A→G	E→G
IQT-1950	P10 – B Alt	4472	NS2B	A→R (A/G)	(I→M)
IQT-1950	P10 – A	5031	NS3	G→R (G/A)	(G→S)
IQT-1950	P10 – B	7083	NS4B	A→W (A/T)	(M→L)
IQT-1950	P10 – B Alt	7409	NS4B	T→C	-
IQT-1950	P10 – B	7505	NS4B	G→T	-
IQT-1950	P10 – A Alt	7505	NS4B	G→K (G/T)	-
IQT-1950	P10 – B Alt	7514	NS4B	T→G	-
IQT-1950	P10 – A	7775	NS5	T→Y (T/C)	-
IQT-1950	P10 – B Alt	8117	NS5	T→C	-
IQT-1950	P10 – B Alt	10362	3′- UTR	A→G	-
IQT-1950	P10 – B	10392	3′- UTR	T→C	-
IQT-1950	P10 – A Alt	10392	3′- UTR	T→C	-
IQT-1950	P10 – B Alt	10392	3′- UTR	T→C	-

a Each passage was performed in duplicate (series A and B); P10 = passage 10; Alt = alternate passage; - = no change.

When either DENV strain was allowed to specialize in C6/36 cells a different mutation distribution emerged. Whereas serial passages in the vertebrate Huh-7 cell line or alternating passages between vertebrate and invertebrate cell lines resulted in mutations distributed throughout the genome (Tables 4 and 5), serial passage in mosquito cells led to a smaller number of consensus mutations that were concentrated within the non structural genes and the 3′ – UTR (Table 6).

**Table 6:** Nucleotide (nt) and amino acid (aa) changes in sequences of C6/36-passaged DENV strains relative to uncloned parent DENV.

Virus	Passage^a^	Cell line	nt position	Region	nt change	aa change
IQT-1950	P10 – A	C6/36	2906	NS1	A→R (A/G)	-
IQT-1950	P10 – A	C6/36	4471	NS2B	T→C	I→T
IQT-1950	P10 – B	C6/36	4471	NS2B	T→C	I→T
IQT-1950	P10 – A	C6/36	5144	NS3	T→Y (T/C)	-
IQT-1950	P10 – A	C6/36	7416	NS4B	A→G	T→A
IQT-1950	P10 – B	C6/36	7416	NS4B	A→G	T→A
IQT-1950	P10 – A	C6/36	10392	3′-UTR	T→Y (T/C)	-
IQT-1950	P10 – B	C6/36	10392	3′-UTR	T→C	-
IQT-1950	P10 – A	C6/36	10551	3′-UTR	G→R (G/A)	-
P8-1407	P10 – A	C6/36	4902	NS3	C→T	-
P8-1407	P10 – B	C6/36	4902	NS3	C→Y (C/T)	-
P8-1407	P10 – A	C6/36	6579	NS4A	T→A	-
P8-1407	P10 – B	C6/36	6579	NS4A	T→W (T/A)	-
P8-1407	P10 – A	C6/36	9179	NS5	G→S (G/C)	(G→A)
P8-1407	P10 – A	C6/36	10560	3′-UTR	T→C	-

a Each passage was performed in duplicate (series A and B). P10 = passage 10.

To eliminate the possibility that mutations in the alternating passage series occurred at the first transition between invertebrate and vertebrate cells due to bottleneck events, virus derived from both cell lines at passage 2 was sequenced. No consensus mutations were observed at that stage. However when progeny derived from the 5^th^ alternating round of replication was sequenced, a number of mutations that were observed in passage 10 were present (data not shown), suggesting that they were not due to bottlenecks early on the cyclic transitions from vertebrate and invertebrate cells but developed over time stochastically or due to positive selection.

### Consensus sequence changes of cloned DENV

Biological clones of both DENV strains were generated by two rounds of plaque purification on Vero cells and their consensus sequences were determined. Comparison to the genomic sequences of the uncloned viruses revealed a number of nucleotide differences (Table 3). The consensus genomic sequences of the 10^th^ serial passage in Huh-7 and C6/36 cells and the final alternating passage (in Huh-7) were compared to the parental cloned DENV sequences. Sylvatic strain P8-1407 acquired 5 synonymous mutations after 10 alternating passages that were distributed throughout the genome; 2 were nucleotide mixtures and 3 were synonymous. When strain P8-1407 was allowed to specialize in Huh-7 cells it also acquired 5 consensus mutations throughout the genome of which none appeared as nucleotide mixtures and 3 were synonymous. After 10 alternating passages, endemic strain IQT-1950 accrued 3 consensus mutations of which 2 were synonymous (Table 7). When the same strain was allowed to specialize in Huh-7 cells it accrued 4 consensus, non-synonymous mutations of which 3 presented as nucleotide mixtures (Table 7).

**Table 7:** Nucleotide (nt) and amino acid (aa) changes in sequences of Huh-7 – passaged and alternating-passaged DENV relative to cloned parent DENV strains.

Virus	Passage^a^	nt position	Region	nt change	aa change
IQT-1950	P10 – A	1539	E	G→A	E→K
IQT-1950	P10 – B	1539	E	G→R (G/A)	(E→K)
IQT-1950	P10 – B Alt	1539	E	G→A	E→K
IQT-1950	P10 – A	2310	E	A→W (A/T)	(I→L)
IQT-1950	P10 – B	4411	NS2B	C→Y (C/T)	(T→I)
IQT-1950	P10 – B Alt	5348	NS3	A→G	-
IQT-1950	P10 – A Alt	6980	NS4B	G→T	-
IQT-1950	P10 – A	7147	NS4B	C→T	T→I
P8-1407	P10 – A	228	C	A→G	-
P8-1407	P10 – B Alt	1905	E	A→R (A/G)	-
P8-1407	P10 – A	2030	E	T→C	I→T
P8-1407	P10 – B	2030	E	T→C	I→T
P8-1407	P10 – A Alt	2883	NS1	A→G	-
P8-1407	P10 – A	4707	NS3	A→G	-
P8-1407	P10 – A Alt	5974	NS3	T→Y (T/C)	(C→R)
P8-1407	P10 – B	7385	NS4B	C→T	T→M
P8-1407	P10 – A	8334	NS5	C→T	-
P8-1407	P10 – A Alt	10400	3′ – UTR	T→C	-
P8-1407	P10 – B Alt	10422	3′ – UTR	G→T	-

a Each passage was performed in duplicate (series A and B); P10 = passage 10; Alt = alternate passage; - = no change.

Serial passage of either cloned DENV strain in C6/36 cells resulted in a similar mutation profile as that observed with the uncloned strains. All mutations were within the nonstructural genes and 3′ – UTR. Specifically, the endemic IQT-1950 strain developed one consensus, non-synonymous mutation, while the sylvatic P8-1407 strain accrued 5 consensus mutations of which one was non-synonymous and one presented as a nucleotide mixture (Table 8).

**Table 8:** Nucleotide (nt) and amino acid (aa) changes in sequences of C6/36-passaged DENV relative to cloned parent DENV strains.

Virus	Passage^a^	nt position	Region	nt change	aa change
IQT-1950	P10 – A	4471	NS2B	T→Y (T/C)	(I→T)
IQT-1950	P10 – B	4471	NS2B	T→Y (T/C)	(I→T)
P8-1407	P10 – A	2883	NS1	A→R (A/G)	-
P8-1407	P10 – B	5390	NS3	A→G	D→G
P8-1407	P10 – B	7305	NS4B	C→T	-
P8-1407	P10 – A	10400	3′- UTR	T→C	-
P8-1407	P10 – B	10422	3′- UTR	G→T	-

a Each passage was performed in duplicate (series A and B); P10 = passage 10; - = no change.

### Patterns of mutation accumulation and distribution

We compared the accumulation of mutations, measured as the mean number of total mutations in each passage series in each virus (N = 2 viruses×3 passage series = 6), in cloned relative to uncloned populations. Cloned viruses accumulated significantly fewer mutations than uncloned viruses in the same passage series (paired t-test, df = 5, P = 0.002). We therefore considered cloned and uncloned viruses separately in subsequent analyses.

To evaluate the impact of passage series on mutation accumulation, we first tested whether the 2 DENV strains used in the study (P8-1407 and IQT-1950) differed in the number of mutations following passage. An unpaired t-test using each passaged virus line (N = 6/virus) showed no difference between viruses in overall number of mutations in either the uncloned (df = 10, P = 0.77) or cloned (df = 10, P = 0.33) populations. We therefore combined data from the two viruses and used an ANOVA to compare total number of mutations among passage series (Table 9). In both uncloned and cloned populations, viruses passaged in C6/36 cells accumulated fewer mutations than those passaged in either Huh-7 cells or in alternation. While these differences were not significant in the cloned population (df = 2, F = 2.13, P = 0.17), in the uncloned population a significant difference among passage series was detected (df = 2, F = 5.4, P = 0.03) and a Tukey-Kramer post-hoc test revealed that viruses passaged in alternation accumulated significantly more mutations than viruses passaged exclusively in C6/36 cells (Table 9). This was particularly striking given that viruses in the alternating passage series experienced twice as many passages as those passaged exclusively in either cell line.

**Table 9:** Overall patterns of DENV mutation accumulation.

Passage Series	Total number of mutations±SE	
	Uncloned viruses	Cloned viruses
HuH-7 only	5.25±0.25^AB^ *	2.45±0.48^A^
Alternating	7.00±0.82^A^	2.00±0.41^A^
C6/36 only	3.75±0.85^B^	1.26±0.63^A^

*: Superscripted letters indicate the results of a Tukey-Kramer post-hoc test. Uncloned and cloned viruses were analyzed separately. Values that do not share a letter are significantly different.

Additionally, we assessed the distribution of the 11 non-synonymous (or non-coding region) mutations that occurred in more than one passage series, hereafter termed convergent mutations (Table 10). Several patterns are evident; first, the number of repetitions of convergent mutations ranged from two to five. Second, three duplicate passage series (cloned IQT-1950 passaged on C6/36, uncloned IQT-1950 passaged on Huh-7 and cloned P8-1407 passaged on Huh-7) shared a single convergent mutation and three duplicate passage series (uncloned IQT-1950 passaged in alternation, uncloned IQT-1950 passaged on C6/36 and uncloned P8-1407 passaged in alternation) shared two convergent mutations. Third, in one case both the cloned and uncloned populations of a virus (IQT-1590) shared a single convergent mutation when passaged exclusively in C6/36 cells. Finally, convergent mutations in the coding region were shared between viruses passaged in Huh-7 alone and those passaged in alternation, or between viruses passaged in C6/36 alone and those passaged in alternation. However no convergent mutations shared between viruses passaged in Huh-7 alone and C6/36 alone occurred in the coding region; the two such mutations were located in the 3′ NCR.

**Table 10:** Summary of non-synonymous mutations and mutations in the non-coding regions (NCR) that occurred in more than one passage series.

Mutation	ENDEMIC: Cloned						ENDEMIC: Uncloned						SYLVATIC: Cloned						SYLVATIC: Uncloned					
	H7-A	H7-B	Alt-A	Alt-B	C6-A	C6-B	H7-A	H7-B	Alt-A	Alt-B	C6-A	C6-B	H7-A	H7-B	Alt-A	Alt-B	C6-A	C6-B	H7-A	H7-B	Alt-A	Alt-B	C6-A	C6-B
G1120A																				X^a^	X^a^	X^x^		
G1539A	X			X				X	X	X														
G1539R		X^a^					X^a^																	
T2030C													X	X										
T4471C					X	X					X	X												
A7416G							X	X																
G9179C																				X		X		
G9179S																					X^b^		X^b^	
T10392C^c^								X	X	X	X	X												
T10400C^c^															X		X			X	X	X		
G10422T^c^																X		X						

a Mutation occurred as a G/A mixture.

b Mutation occurred as a G/C mixture.

c Mutation present in the 3′-NCR of the genome.

Abbreviations: H7-A, H7-B – Huh-7 – passaged DENV; C6-A, C6-B – C6/36-passaged DENV; Alt-A, Alt-B – alternate-passaged DENV.

## Discussion

We hypothesized that DENV evolution is constrained by the requirement for alternating replication in highly divergent vertebrate hosts and invertebrate vectors. The trade-off hypothesis predicts that releasing DENV from alternating host replication by repeated passages in a single host or cell line (either vertebrate or invertebrate) will facilitate faster adaptation to that cell line. The hypothesis also predicts that adaptation to a given host or cell line will be specific and will result in fitness declines in other hosts or cell lines. As a consequence of the previous two processes, the hypothesis predicts that genetic change (particularly nonsynonymous mutations) of virus lines passaged in a single host type will be more rapid that lines passaged in alternating hosts.

To evaluate these hypotheses, we passaged DENV viruses 10 times, either continuously or alternately, in vertebrate and invertebrate cells. Moreover, we initiated these passage series with either genetically homogenous (biologically cloned) virus populations or heterogeneous, uncloned populations. The consensus genomic sequences of the experimentally evolved viruses were determined and their fitness was measured by multicycle replication kinetics (replication curves) on both the cell type in which the single-cell line virus had been passaged, as well as in the bypassed cell line and a novel cell type that had not been used previously in the experiment.

Our results from uncloned DENV were generally consistent with the hypothesis that releasing arboviruses from an alternating replication cycle results in the acquisition of higher fitness for the retained host cell and loss of fitness in the bypassed cell. Cloned viruses, in contrast, showed fitness gains in all cell types following passage, irrespective of the passage regimen. A possible explanation for this discrepancy in the patterns of adaptation in uncloned and cloned virus populations is the genetic bottleneck effect of the biological cloning: randomly selected mutations are often deleterious [55]. Evidence from bacteriophage φ6 [56], VSV [57],[58], foot-and-mouth-disease virus (FMDV) [59] and EEEV [10] suggests that repeated plaque-to-plaque transfers (≥40) result in fitness losses caused by genetic bottlenecks and drift. Although cloned viruses in this study were subjected to a limited number of bottleneck events (2 plaque purification events), their consensus sequences did differ from those of the uncloned viruses, thus the general increase in fitness that occurred following passage of any type may reflect selection for both an increase in overall fitness as well as adaptation to specific cell substrates. Previous studies have indicated that starting fitness can affect RNA virus adaptation outcomes [51],[58],[60],[61],[62],[63] and that viruses may acquire both host cell-specific and amphi-cell-specific adaptations during passage [64]. Thus, in the following discussion we use only the data from uncloned viruses to evaluate the predictions of the trade-off hypothesis for changes in fitness.

The hypothesis that adaptation to a given host cell (Huh-7 or C6/36) is specific and results in fitness declines in the bypassed cell line or novel host cells (Vero cells), was generally supported. As predicted, both endemic and sylvatic uncloned viruses allowed to specialize in Huh-7 or C6/36 cells gained fitness in that cell line. Also as predicted, viruses passaged in a single cell line lost fitness in the bypassed cell line. However, fitness increases in the uncloned sylvatic DENV passaged exclusively in mosquito cells partially contradicted the tradeoff hypothesis in that fitness also increased in Vero cells (Table 2). Thus, our Huh-7 or C6/36 cell data are consistent with the hypothesis that adaptation to a given host or cell is specific and results in fitness declines in the bypassed cell line, or in other hosts or cell (Vero cells). However, inconsistent with the hypothesis, we observed significant fitness increases by both cloned endemic and sylvatic DENV viruses upon evaluation in Vero cells. Viruses passaged in alternation showed no change in fitness on either cell type. Patterns of fitness on a novel cell type (Vero) were more complex. While passage in Huh-7 cells reliably led to a decrease in fitness in Vero cells, passage in C6/36 resulted in variable changes in fitness (increases and decreases).

A third prediction of the trade-off hypothesis is that DENV passaged in a single host cell type will accrue more mutations in its consensus sequences, especially nonsynonymous changes, compared to DENV passaged in alternation between two different cell types. As predicted, viruses passaged in a single host cell line accumulated more nucleotide and amino acid changes in their consensus sequences, compared to viruses that replicated in alternating invertebrate-to-vertebrate host cell passages. Overall, 45% and 50% of the mutations generated within the uncloned and cloned viruses, respectively, were non-synonymous. Although these ratios are lower that random expectations, they are considerably higher than what is observed in nature, where the vast majority of nucleotide substitutions are synonymous, revealing purifying selection as the major force shaping arbovirus evolution [65]. Nonetheless, several non-synonymous mutations occurred in multiple passage series within the same passage regime and/or alternate passage regimes, a pattern that strongly suggests convergent evolution via positive selection. Similar observations were described previously, where serial passage of DENV-3 in Vero or C6/36 cells resulted in common amino acid changes that were repeatedly selected in a given cell passage series [66].

Exclusive DENV passage in vertebrate cells or alternating host cell passages led to the emergence of a qualitatively and quantitatively different mutation spectrum than exclusive passage in mosquito cells. Whereas serial passages in the vertebrate Huh-7 host cell or alternating passages between vertebrate and mosquito cells resulted mutations distributed throughout the genome, serial passage in mosquito cells led to mutations located exclusively within the non-structural protein genes and 3′ – UTR. Moreover the rate of mutation accumulation was significantly slower for viruses passaged in C6/36 cells than for those passaged in alternation. These observations support the notion that positive selection exerts a greater influence within the vertebrate host. Previous observations by Chen [67] based on similar experiments and limited consensus sequence of E and NS1 genes provide further support that mosquito cells have a minimal effect on DENV evolution. Reverse genetic studies are needed to examine the contribution of the individual mutations to fitness.

An inherent limitation of utilizing consensus sequences to monitor evolution of viral populations is that they only reflect the majority nucleotide at any given position of the viral genome and do not represent the spectrum of mutations present. Minority mutant populations, characteristic of RNA virus quasispecies, can be masked [68]. Our data indicate the presence of mixed populations at several nucleotide positions. However, it is possible that other mutations that may also play a role in DENV adaptation were present (at lower frequencies) but remained undetected by sequencing of the RT-PCR amplicons. Recent studies of West Nile (WNV) and St. Louis encephalitis viruses (SLEV) demonstrate that the diversity of the quasispecies population can differ in vertebrate hosts and invertebrate vectors [69],[70],[71] and that these differences can affect viral virulence [72]. These studies suggest that viruses passaged in mosquitoes/mosquito cells gain diversity more rapidly than viruses passaged in vertebrates/vertebrate cells. Thus, future studies of DENV should consider the impact of host alternation on the evolution of quasispecies diversity as well as consensus sequence changes. In fact, the continued advances in the efficiency, speed and cost of DNA sequencing including ‘deep sequencing’ can efficiently sequence a large number of RNA viral genomes. This could provide complete information on the quasispecies ensemble.

Understanding the forces that shape DENV evolution and the extent these forces play in the observed shifts to higher virulence in human infections is important in developing effective countermeasures, especially in the absence of a licensed vaccine or antiviral therapies to control the spread of a DENV pandemic.

## Methods

### Cell cultures

Monolayer cultures of Huh-7 cells (clone JTC-39 obtained from the Japanese Health Sciences Foundation, Osaka) and Vero cells (obtained from American Type Culture Collection, Bethesda, MD) were grown at 37°C in Dulbecco's minimal essential medium (DMEM) (4.5 g/L D-Glucose) with 10% heat-inactivated fetal bovine serum (FBS) and 1% penicillin/streptomycin. C6/36 mosquito cells (a generous gift from Ilya Frolov) were grown at 32°C in Dulbecco's minimal essential medium (DMEM) (4.5 g/L D-Glucose) with 10% heat-inactivated FBS, 1% penicillin/streptomycin and 1% tryptose phosphate broth (TPB).

### Viruses and sequencing

To gain insights into potential differences in adaptive constraints on ancestral and derived lineages of DENV, we utilized two different strains of DENV-2 in this study: low passage sylvatic P8-1407 and endemic IQT-1950 DENV-2 (passaged, but uncloned) strains were obtained from the UTMB World Reference Center for Emerging Viruses and Arboviruses and amplified once in C6/36 mosquito cells to achieve titers of ∼7 log_10_ focus forming units (ffu)/ml. Sylvatic P8-1407 was isolated from a sentinel monkey in Malaysia in 1970 and endemic IQT-1950 was isolated from a viremic human in Peru in 1995 (see [42] for passage history of strains). In order to establish homogeneous virus populations for our study, biological clones of each strain were generated by two rounds of plaque purification on Vero cells. To ensure at that the ‘picked’ visible clonal DENV populations were not representing a minority population with unique phenotypic and morphologic characteristics, each visible plaque was marked. Then the agarose plug was carefully removed without disturbing the monolayer and each monolayer was fixed with 1 ml of 1∶1 methanol∶acetone for 20 minutes at room temperature. After removal of the fixative, the plates were allowed to air-dry, and infected cells were detected by FFA as described below. Selected clones were representative of the majority of clones present in the well (Table 11). Each of the selected clones were then propagated once in C6/36 cells in order to generate stocks for the passage and replication kinetics studies. Each virus stock was quantified by FFA assay on C6/36 and Huh-7 cell lines then aliquoted and stored at −80°C.

**Table 11:** Morphological plaque characteristics of cloned parent DENV.

	Sylvatic strain P8-1407			Endemic strain IQT-1950		
	Plaques^a^ (AD)^b^	IHC^c^ (AD)^b^	%^d^	Plaques^a^ (AD)^b^	IHC^c^ (AD)^b^	%^d^
Well 1	32 (1 mm)	5 (0.3 mm)	86.5	39 (2 mm)	4 (0.4 mm)	90.7
Well 2	30 (1 mm)	3 (0.3 mm)	91.0	46 (2 mm)	5 (0.4 mm)	90.2

a Number of DENV plaques visible by neutral red staining on 6-well plates of Vero cells.

b AD – Average diameter of plaques in millimeters (mm).

c Number of DENV plaques, in addition to the number of visible by neutral red plaques present in the same well, after development of IHC.

d Percent of plaques visible, per well.

Genomic viral RNA (vRNA) was isolated with the Qiagen viral RNA isolation kit (Qiagen, Valencia). The 5′ and 3′- terminus sequences of the specific genomes were determined by ligation and sequencing as described by Mandl et al. [73]. Overlapping cDNA fragments and amplicons of these viruses were generated using virus-specific primer pairs and one-step RT-PCR (Roche Diagnostics, Indianapolis). Amplified sequences were gel purified and automated sequencing with virus-specific sequencing primers for both strands.

### Dengue virus infections

Three different types of passage regimens were performed for each of the 4 DENV populations (P8-1407 and IQT 1950, cloned and uncloned): (i) serial, exclusive passage in Huh-7 cells; (ii) serial, exclusive passage in C6/36 cells; and (iii) alternating passage between Huh-7 and C6/36 cells. Each strain was passaged in each regimen in duplicate. Each alternating cycle constituted a round of DENV infections between C6/36 and Huh-7 cells with Huh-7 being the concluding cell line of the cycle. In order to eliminate temperature sensitivity as a factor in the outcomes, all infected cell cultures were incubated at 32°C. Infected cell culture supernatants were harvested at 4 days post infection to achieve maximum titers. Cell-free cell culture supernatants were diluted to a multiplicity of 0.01 ffu/ml (based on titers obtained on C6/36 cells) to initiate subsequent infections. A low multiplicity of infection (MOI) was maintained to minimize the influence of defective interfering (DI) particles. To assess variance in outcomes and to identify common mutations arising due to positive selection, duplicate parallel passage series were performed for each treatment.

### Focus forming assay (FFA)

Ten-fold serial dilutions of virus in MEM supplemented with 2% FBS and antibiotics (Invitrogen, Carlsbad, CA), were inoculated in duplicate to confluent C6/36 cell monolayers attached to 24-well Costar® plates and incubated for one hour with periodic gentle rocking to facilitate virus adsorption at 37°C. Subsequently, wells were overlaid with 1 ml of 0.8% methylcellulose (Sigma-Aldrich, St. Louis, MO) diluted in warm Optimem (Invitrogen) supplemented with 2% FBS, antibiotics and 1% (w/v) L-glutamine, then incubated undisturbed for 4 days at 28°C. The methylcellulose overlay was then aspirated and cell monolayers were rinsed once with PBS at pH 7.4 (Invitrogen) then fixed with a mixture of ice-cold acetone and methanol (1∶1) solution and allowed to incubate for 30 minutes at room temperature. The fixation solution was aspirated and plates were allowed to air dry. Plates were washed thrice with PBS supplemented with 3% FBS, followed by hour-long incubation with a dengue-specific mouse ascitic fluid. Plates were washed thrice followed by hour-long incubation with a secondary antibody conjugated to horseradish peroxide (KPL, Gaithersburg, MD). Detection proceeded with the addition of AEC substrate (ENZO Diagnostics, Farmingdale, NY) prepared according to vendor instructions. Antigen stained cells were counted against a white background and viral titers were recorded as the reciprocal of the highest dilution where adequate plaques were detected (greater than 20 but lower than 90) and expressed as log_10_focus forming units per ml (ffu/ml).

### Replication kinetics

Multistep replication curves were performed to measure virus fitness in each of three cell types: Vero, mosquito C6/36 or Huh-7. Because of the large number of samples generated, all replication curves could not be titered in a single assay. Consequently, a single virus population passaged serially in one cell type was titered together with the same population passaged in alternation and with the parent. For example, P8-1407 (uncloned) that had been passaged only in Huh-7 cells was titered in the same assay as P8-1407 (uncloned) passaged in alteration and the parental P8-1407 (uncloned) virus. P8-1407 (uncloned) that had been passaged only in C6/36 cells was titered in a separate assay along with P8-1407 (uncloned) passaged in alteration and the parental P8-1407 (uncloned) virus. This subdivision of assays meant that fitness of viruses passaged only in Huh-7 or C6/36 cells could not be compared statistically (see below), a limitation we considered acceptable since the question being addressed was whether virus passaged in a single cell type would differ from virus passaged in alternation and not whether viruses passaged in different single cell types differed from each other.

Dishes (30 mm in diameter) containing confluent Vero, mosquito C6/36 or Huh-7 cell monolayers were prepared and cell number was determined. Viruses were diluted in MEM supplemented with 5% FBS, 2 mM L-Glutamine, 1% NEAA, and 50 mg/ml penicillin/streptomycin (Invitrogen), at an MOI of 0.1 ffu/cell (based on titers obtained on C6/36 cells) are infected simultaneously in triplicate. Infected dishes were incubated for one hour with periodic gentle rocking to facilitate virus adsorption at 37°C. Viral inocula were removed and cell monolayers were washed 3 times with PBS to remove un-adsorbed virus. Two ml of complete cell media (MEM supplemented with 5% FBS, 2 mM L-Glutamine, 1% NEAA, and 50 mg/ml penicillin/streptomycin) were then added and cells were incubated at 28°C or 37°C for the mosquito or mammalian cell lines respectively. At various times (0, 1, 2, 4, and 6 days) post infection, virus from individual dishes were harvested, purified by low speed centrifugation, and stored at −80°C. Upon completion of the experiment, virus samples representing the collected time points were assayed by FFA to determine virus titers. Virus yield at each timepoint was recorded as log_10_ ffu/cell, the ratio of the total amount of virus present in the sample by the number of cells originally infected.

### Statistical analysis

Repeated-measures ANOVA [74] was used to compare the impact of passage regimen on replication curves of each of the four virus populations in each of three cell types (Vero, Huh-7 and C6/36). For reasons described above, a given DENV population serially passaged in a single cell line was compared only to the matched population passaged in alternation and to the parent. All comparisons revealed a significant effect of passage regimen (df = 4, P<0.001 for each comparison), a significant effect of day (df = 4, P<0.001 for each comparison), and a significant interaction between the two factors (df = 16, P<0.001 for each comparison). A Tukey-Kramer post-hoc test was used to detect differences among passage regimens within each experimental group. Since this resulted in a large number of comparisons, conclusions were only drawn from consistent patterns of significant difference revealed in multiple comparisons.
